# Developmental Changes in the *in Vitro* Activated Regenerative Activity of Primitive Mammary Epithelial Cells

**DOI:** 10.1371/journal.pbio.1001630

**Published:** 2013-08-13

**Authors:** Maisam Makarem, Nagarajan Kannan, Long V. Nguyen, David J. H. F. Knapp, Sneha Balani, Michael D. Prater, John Stingl, Afshin Raouf, Oksana Nemirovsky, Peter Eirew, Connie J. Eaves

**Affiliations:** 1 Terry Fox Laboratory, British Columbia Cancer Agency, Vancouver, British Columbia, Canada; 2 Cancer Research UK Cambridge Research Institute, Li Ka Shing Centre, Cambridge, United Kingdom; 3 Department of Immunology and The Regenerative Medicine Program, Manitoba Institute of Cell Biology, University of Manitoba, Winnipeg, Manitoba, Canada; 4 Departments of Medical Genetics, Medicine, and Pathology and Laboratory Medicine, University of British Columbia, Vancouver, British Columbia, Canada; Helmholtz Zentrum Muenchen, Germany

## Abstract

Many normal adult tissues contain rare stem cells with extensive self-maintaining regenerative potential. During development, the stem cells of the hematopoietic and neural systems undergo intrinsically specified changes in their self-renewal potential. In the mouse, mammary stem cells with transplantable regenerative activity are first detectable a few days before birth. They share some phenotypic properties with their adult counterparts but are enriched in a subpopulation that displays a distinct gene expression profile. Here we show that fetal mammary epithelial cells have a greater direct and inducible growth potential than their adult counterparts. The latter feature is revealed in a novel culture system that enables large numbers of *in vitro* clonogenic progenitors as well as mammary stem cells with serially transplantable activity to be produced within 7 days from single fetal or adult input cells. We further show that these responses are highly dependent on novel factors produced by fibroblasts. These findings provide new avenues for elucidating mechanisms that regulate normal mammary epithelial stem cell properties at the single-cell level, how these change during development, and how their perturbation may contribute to transformation.

## Abstract 2

Mouse fetal mammary cells display greater regenerative activity than do adult mammary cells when stimulated to proliferate in a new system that supports the production of transplantable mammary stem cells *ex vivo*.

## Author Summary

Many adult tissues are maintained by a rare subset of undifferentiated stem cells that can self-renew and give rise to specialized daughter cells that have a more limited regenerative ability. The recent identification of cells in the fetal and adult mammary gland that display the properties of stem cells provides a foundation for investigating their self-renewal and differentiation control. We now show that these stem cell properties can be elicited from single mouse mammary cells placed in 3D cultures if novel factors produced by fibroblasts are present. Moreover, a comparison of the clonal outputs of fetal and adult mammary cells in this *in vitro* system shows that the fetal mammary cells have superior regenerative activity relative to their adult counterparts. The ability to activate and quantify the regenerative capacity of single mouse mammary epithelial cells *in vitro* sets the stage for further investigations of the timing and mechanisms that alter their stem cell properties during development, the potential relevance of these events to other normal epithelial tissues, and how these processes might be involved in the genesis of breast cancer.

## Introduction

The regenerative properties of individual cells within the mammary gland were first indicated by the retrovirally marked clonal outgrowths shown to develop from mouse mammary tissue fragments transplanted into the cleared mammary fat pad [1],[2]. More recently, we and others have demonstrated that individual cells isolated from the adult mammary gland are capable of regenerating a complete new gland when transplanted in the same type of assay and most of these are confined to a distinct subset of cells with basal (CD24^+^/EpCAM^+^CD49f^+^) features [3]–[5]. The regenerated mammary glands thus produced contain the same spectrum of cell types that are present in the adult mammary gland. These include progenitor cells (referred to as colony-forming cells, or CFCs) with a luminal (CD24^++^/EpCAM^++^CD49f^low/−^) phenotype and other cells with either a luminal or basal phenotype that are considered to be differentiated because they lack proliferative ability. In addition, the structures produced *in vivo* contain cells with the same transplantable regenerative activity as the original parental input cell. The latter are thus referred to operationally as mammary repopulating units, or MRUs, based on the method used to detect them. MRUs can be quantified by limiting dilution analysis (LDA) of their ability to regenerate large branched glandular structures when transplanted into the cleared fat pad of prepubertal mice [3],[4]. This MRU assay has now been widely used to investigate mechanisms that regulate normal adult mammary stem cell differentiation and growth control [6], as well as the effects of various mutations that contribute to the genesis of breast cancer [7].

Previous studies of the development of the mouse mammary gland have shown that the first elements appear on embryonic day 11 (E11) as placodes of specified ectoderm. The cells in these placodes then expand in number and invaginate into the underlying mesenchyme to develop primordial branched structures that, just before birth, are found to contain cells detectable as individually transplantable MRUs [8],[9]. Interestingly, these MRUs, like their adult counterparts, belong to a subset of cells that are CD49f^+^ but also have phenotypic and transcriptional differences [8]. However, whether fetal and adult MRUs have different growth and self-renewal properties, as described for stem cell populations in some other tissues [10],[11], is not known. The higher self-renewal activity characteristic of these fetal tissues has been attributed to intrinsic molecular mechanisms operating within the stem cells, themselves, albeit in response to environmental cues, and include transcriptional regulators such as *Sox17* [12] and Lin28 [13],[14] in hematopoietic stem cells, and *Hmga2* in neural [15] and hematopoietic stem cells [14].

The investigation of such differences requires the availability of a robust system in which the maximum self-renewal/regenerative activity of the stem cells of interest can be elicited and quantified. Such a system has not yet been developed and validated for mammary epithelial stem cells, although a variety of candidate elements have been reported. One of these is Matrigel, a laminin-rich tumor extract that has been widely used to support mammary epithelial cell growth *in vitro* [3],[8],[16]–[18]. Interestingly, adult basal mammary cells have been found to display increased clonogenicity in Matrigel in the presence of Wnt3a and also retain some MRU activity when serially passaged under these conditions [18]. There is also evidence that the number of MRUs detected in transplants of both basal cells and luminal cells of mouse origin is enhanced when Matrigel is injected together with the cells [5],[19]. Some latent activity or enhanced detection of MRUs in basal and even luminal mammary cells has also been revealed in cells exposed to a Rho-associated kinase inhibitor (ROCKi) [20]. We now report the superior ability of fetal mammary epithelial cells to produce MRUs and CFCs as compared to their adult counterparts when they are activated in a Matrigel-based clonal culture system that requires distinct, but as yet unidentified factors produced by fibroblasts.

## Results

### The Phenotype But Not the Ratio of MRUs to CFCs in the Mammary Epithelium Changes Between E18.5 and Adulthood

**Figure 1:**
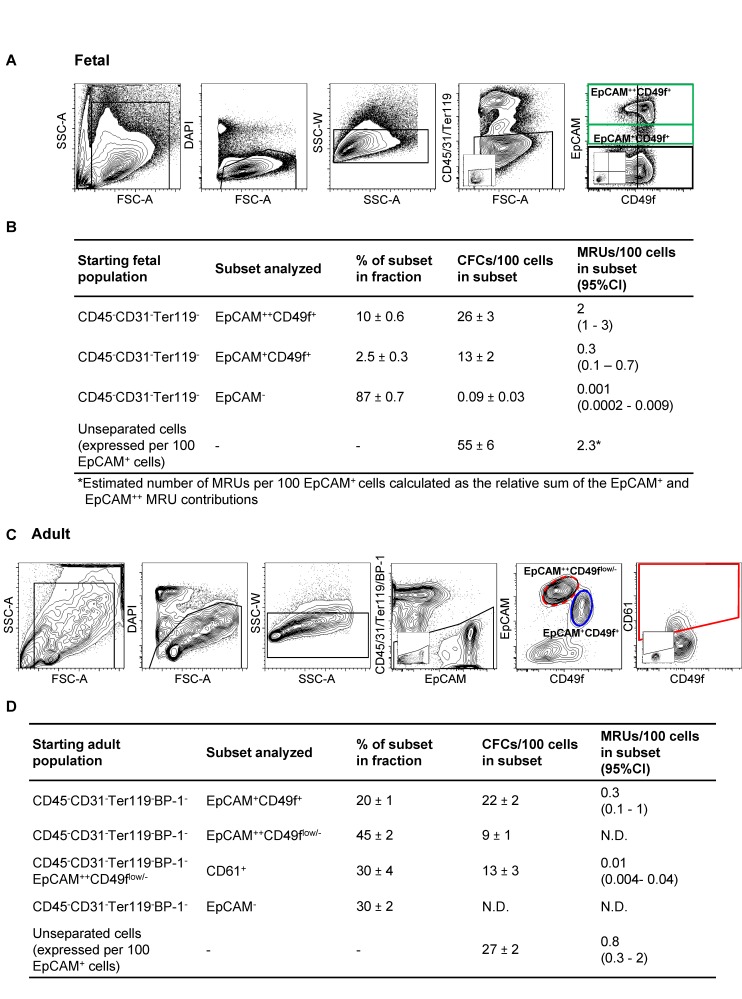
Representative frequencies of phenotypes, CFCs, and MRUs in E18.5 fetal and adult mammary tissue. (A) Representative gating and final FACS profiles of viable subsets of single cells isolated from dissociated E18.5 mammary glands after exclusion of hematopoietic (CD45^+^ and Ter119^+^) and endothelial (CD31^+^) cells. Embedded images are negative controls. (B) Frequency of total cells, CFCs, and MRUs measured in the indicated subsets and in suspended but not further separated fetal mammary (EpCAM^+^) cells. (C) Representative gating and final FACS profiles of viable subsets of single cells isolated from dissociated 8–12-wk-old adult mammary glands after exclusion of hematopoietic (CD45^+^ and Ter119^+^), endothelial (CD31^+^), and stromal (BP-1^+^) cells. A fluorescence-minus-one control was used to set the gate used to identify CD61^+^ cells. (D) Frequency of total cells, CFCs, and MRUs measured in the indicated subsets and in unseparated suspensions of adult mammary epithelial (EpCAM^+^) cells.

**Figure 2:**
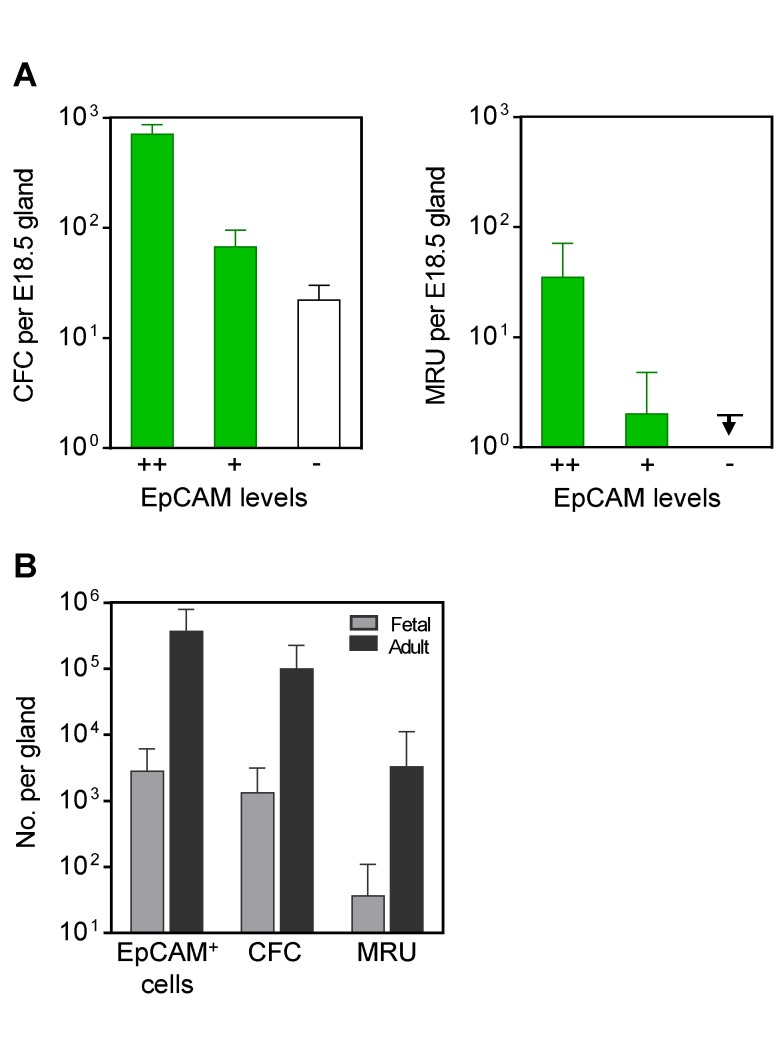
Quantification of the CFC and MRU content of fetal compared with adult mammary cells. (A) Distribution of fetal MRUs and CFCs according to their expression of EpCAM. CFC values are the mean ± SEM for ≥4 experiments. MRU and 95% CI values were determined by LDA. The arrow indicates that MRUs, if present in the EpCAM^−^ fraction, were below the limit of detection (indicated by the line; for details, see Table S4). (B) Comparison of CFC and MRU content of cells from >15 and 2 adult no. 4 glands, respectively, and >50 fetal glands. CFC values are geometric means. Error bars for MRU and CFC values show 95% CI.

In a first series of experiments, we determined the number and phenotype of MRUs and CFCs in the mammary rudiment present in the E18.5 female C57Bl/6 (B6) embryo. E18.5 was chosen because it is the earliest time during the development of the mammary gland when MRUs are reproducibly detected in numbers sufficient for their characterization [8]. In these experiments we also found that antibody staining of EpCAM^+^ and CD49f^+^ cells in enzymatically dissociated viable single-cell suspensions of dissected E18.5 fetal glands [from which the associated endothelial (CD31^+^) and blood (CD45^+^ and Ter119^+^) cells had been excluded] revealed the presence of three major subpopulations (Figure 1A). These consisted of an EpCAM^++^CD49f^+^ fraction and an EpCAM^+^CD49f^+^ fraction of the fetal mammary epithelial cells and an EpCAM^−^ fraction that is variably CD49f^+^ and contains the associated stromal cells. Assessment of the MRU and CFC content of the fetal mammary gland showed that the majority of both of these functionally defined primitive cell types are present in the EpCAM^++^CD49f^+^ subset (Figures 1B and 2A, Tables S1, S2, S3, S4), as determined using optimized assay conditions for both of these (Figure S1A–D). Interestingly, the fetal gland appears to lack entirely the large population of EpCAM^++^CD49f^low/−^ luminal cells present in the mammary gland of 8–12-wk-old adult virgin female mice (Figure 1A). In addition, the majority of the CD49f^+^ cells in the fetal gland are EpCAM^++^, whereas the majority of the CD49f^+^ cells in the adult gland are EpCAM^+^ (Figure 1B and D).

Calculation of MRU and CFC frequencies as a function of the number of EpCAM^+^ cells tested allows comparisons to be made that relate exclusively to the gland itself or derived populations, even when unseparated fractions are being evaluated. Such a comparison of the MRU and CFC frequencies and content in the E18.5 fetal (Figure 1B) and adult (Figure 1D) mammary gland showed that the ratio of their numbers relative to one another is already set at approximately the same value as that characteristic of the adult virgin gland, although the total size of these populations as well as the size of the gland as a whole (total number of EpCAM^+^ cells) undergo an expansion of ∼100-fold over the intervening 4-mo period (Figure 2B).

### Fibroblast-Containing Matrigel Cultures Support the Production of Large Numbers of CFCs and MRUs From Both Fetal and Adult Mammary Cells

**Figure 3:**
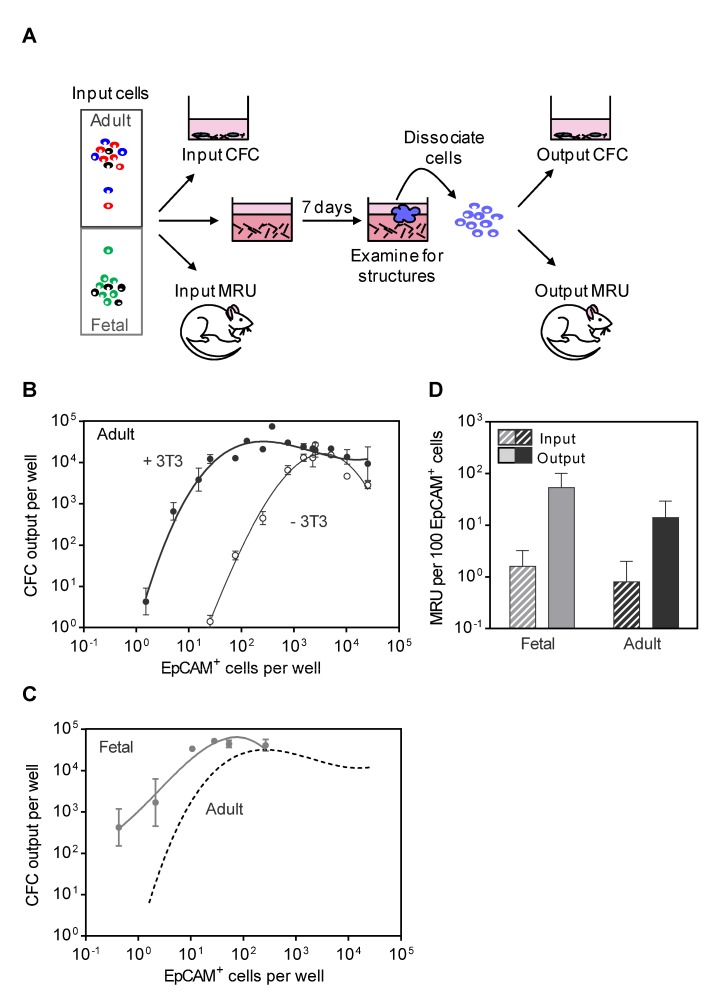
Production of MRUs and CFCs in 7-d Matrigel cultures of adult and fetal mammary cells. (A) General experimental design. Mammary cells were added to 50 µl of solidified Matrigel (±2.5×10^4^ irradiated 3T3 fibroblasts) in 200 µl of medium and incubated for 7-d. Each well was then examined for the presence of one or more visible structures and the contents then fixed and stained or dissociated into a suspension of viable single cells to perform FACS, CFC, or MRU assays for comparison with corresponding starting (input) values. (B) CFC outputs from 7-d Matrigel cultures of unseparated adult mammary cells as a function of the input cell number (expressed as the number of input EpCAM^+^ cells) and the addition of irradiated fibroblasts. (C) CFC outputs from 7-d cultures of unseparated fetal cells as a function of the input cell number (expressed as the number of input EpCAM^+^ cells). Comparison of the corresponding relationship for adult cells (dotted line redrawn from (B)) shows a ∼5-fold higher CFC output by the fetal cells. (D) Comparison of increased numbers of MRUs obtained from 7-d Matrigel cultures of fetal and adult mammary cells (values determined by LDA as described in Tables S1, S2, S3, S4, S5, S6 and expressed relative to 100 EpCAM^+^ cells).

To compare the regenerative properties of fetal and adult mammary epithelial cells (Figure 3A), we first undertook a preliminary survey of culture conditions that would optimize the *production* over a period of 7 d of adult CFCs measured in secondary 2D CFC assays. These experiments showed superior outputs of CFCs were obtained when cells were incubated in 3D Matrigel cultures as compared to 2D adherent CFC assay conditions (unpublished data). Additional experiments showed that reduced O_2_ conditions had no effect on CFC *production*, regardless of the input cell source (Figure S2A) and added ROCKi had a slight enhancing effect only in cultures initiated with adult luminal cells (Figure S2B).

Cultures initiated with varying inputs of unseparated adult cells showed a marked cell dose–response relationship with linearly increasing outputs of CFCs at cell input numbers below 10^3^ EpCAM^+^ cells per 250 µl. But cultures initiated with more than 5×10^3^ EpCAM^+^ cells became inhibitory (Figure 3B). In addition, we found that the addition of irradiated 3T3 cells to cultures initiated with low numbers of adult EpCAM^+^ cells greatly enhanced the number of CFCs produced in the 7-d Matrigel cultures (∼1,000-fold). Fetal mammary cells placed in the same culture conditions showed a similar input cell dose–dependent increase in CFC output, but much higher CFC outputs were obtained (∼50- to 100-fold) from the same number of EpCAM^+^ input cells as compared to their adult counterparts (Figure 3C). We then assessed the ability of this culture system to support the production of MRUs. When nonsaturating numbers of input fetal or adult mammary cells were used to initiate the cultures, MRU numbers were also increased (Figure 3D, Tables S5 and S6). Moreover, the output of MRUs from the same number of EpCAM^+^ cells was again higher (∼4-fold) for the fetal as compared to adult cells.

These findings indicate the ability of fibroblast-containing Matrigel cultures to support the generation within 7 d of expanded populations of MRUs and CFCs and show that the output of both these cell types is greater for fetal mammary epithelial cells than for their adult counterparts.

### 3T3 Cells Enhancement of CFC and MRU Production in Matrigel Cultures Involves Novel Factors

**Figure 4:**
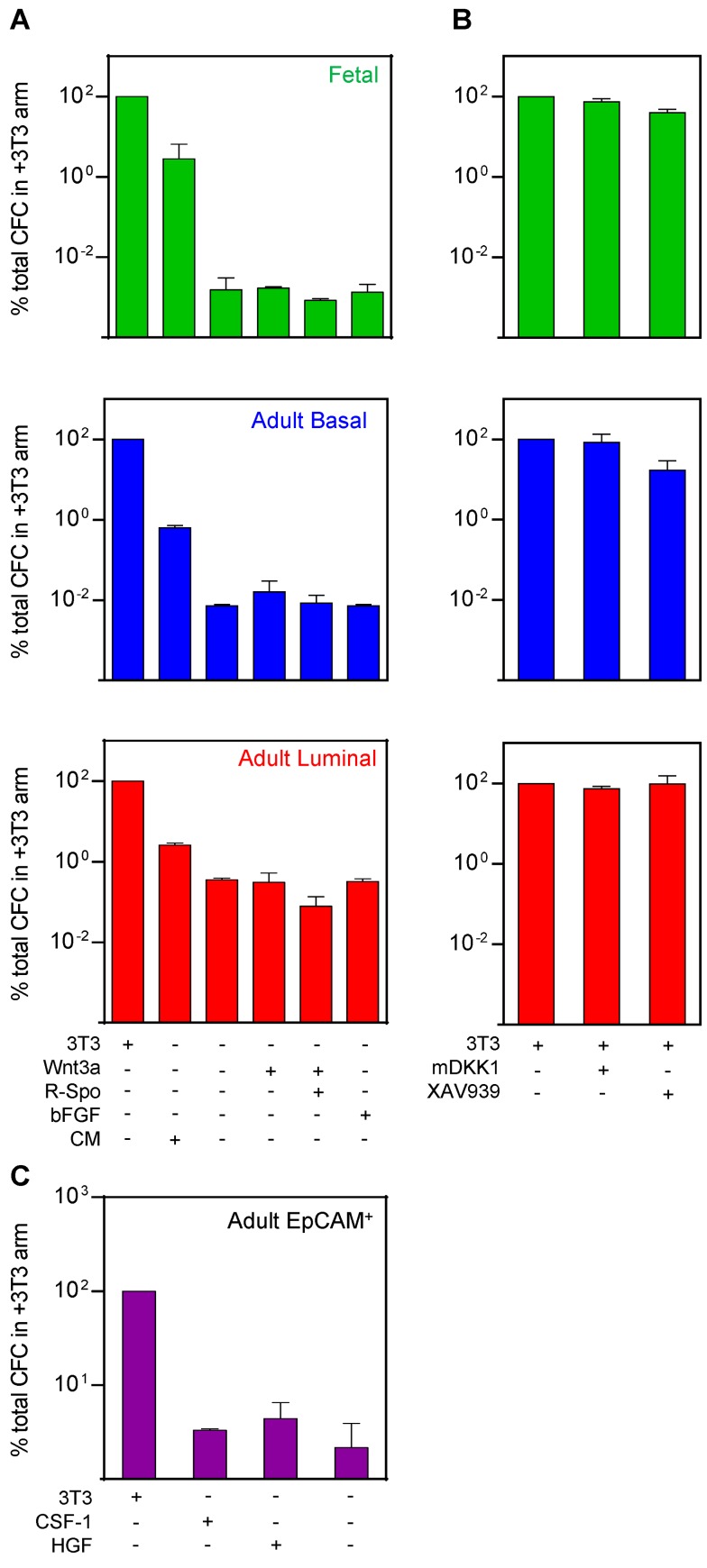
The effect of 3T3 cell CM and selected factors on fetal and adult basal and luminal mammary cell production of CFCs. CFC assays were performed on 7-d cultures of 30 EpCAM^++^ fetal cells (green), 60 EpCAM^+^CD49f^+^ adult basal cells (blue), 100 EpCAM^++^CD49f^low/−^CD61^+^ adult luminal cells (red), and 300 adult EpCAM^+^ cells (purple) using various additives to determine their effects on CFC outputs. (A) Comparison of the effect of 80% 3T3 cell CM, 160 ng/ml Wnt3a±400 ng/ml R-Spondin 1 (R-Spo), or 16 ng/ml bFGF relative to added 3T3 cells (set = 100%). The concentrations shown are the final concentrations in the 250 µl cultures. Results are pooled from 3–6 experiments. The difference in CFC output between CM and no added 3T3 cells was significant for cultures initiated with all types of cells (*p*<0.05, one-way ANOVA with Bonferroni's multiple comparison test). (B) Effect of Wnt pathway inhibitors: XAV939 (0.8 µM for adult, 4 µM for fetal) or mDKK1 (160 ng/ml) in cultures with irradiated 3T3 cells (set = 100%). Results are pooled from 3–9 experiments. Only the effect of added XAV939 was significant, and only for basal cells (*p* = 0.04, one-way ANOVA with Bonferroni's multiple comparison test). (C) Effect of 40 ng/ml HGF and 16 ng/ml CSF-1 on adult EpCAM^+^ cells. The difference in CFC output between added HGF or CSF-1 and no added 3T3 cells is not significant (*p*>0.99, one-way ANOVA with Bonferroni's multiple comparison test).

Experiments using a transwell system suggested that the enhancing effect of the added 3T3 cells is mediated, at least in part, by soluble factors (unpublished data). Subsequent experiments showed that the effects of the irradiated 3T3 cells could be partially replaced by addition of 80% 3T3-cell conditioned medium (CM) (Figure 4A and B). However, the activity in the CM could not be replaced by the addition of various reported single “niche” elements, including basic fibroblast growth factor (bFGF), colony-stimulating factor 1 (CSF-1), or hepatocyte growth factor (HGF) tested at concentrations previously found to stimulate mammary cells or other cell types (16 ng/ml bFGF [21], 16 ng/ml CSF-1 [22], and 40 ng/ml HGF [23],[24], Figure 4A and C). The activity produced by the 3T3 cells also appears to be different from Wnt3a [18], as neither 160 ng/ml of Wnt3a alone or plus 400 ng/ml R-spondin 1 [25],[26] even partially mimicked the effect of 3T3 cells in our cultures (Figure 4A). In addition, the effect of the added 3T3 cells could only be minimally inhibited, and only on adult basal cells (*p* = 0.04), by the addition of one of two Wnt pathway inhibitors [18],[27] tested (XAV939 at 0.8 µM, Figure 4B).

### Immunophenotypic Characterization of the Cells Present in Mammary Structures Produced From Single Purified Fetal, Adult Basal, and Luminal Cells

**Figure 5:**
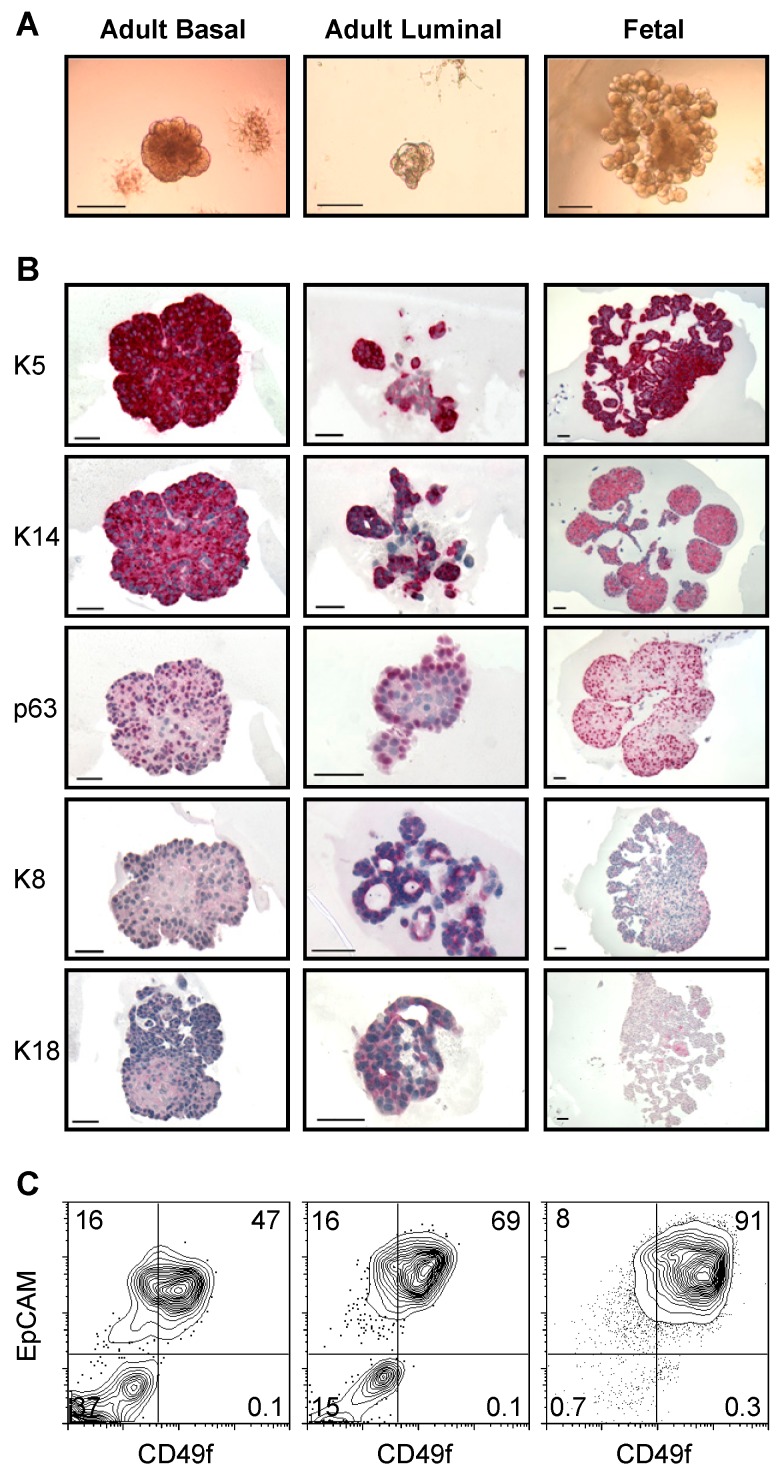
Morphology and cellular composition of structures generated in 7-d Matrigel cultures of single fetal and adult mammary cells. (A) Gross appearance of representative structures from each of the three types of input cells tested. The scale shown indicates 30 µm. (B) Representative photomicrographs of sectioned structures stained with antibodies against the markers shown. Scale, 50 µm. (C) Flow cytometric analysis of dissociated PI^−^ cells obtained from pooled harvests of cultures initiated with the same types of cells.

The above findings showed that readily detectable numbers of CFCs had been generated over a period of 7 d in cultures initiated with suspensions estimated to contain a single EpCAM^+^ cell (Figure 3B and C). In such cultures, the discrete structures formed were readily visualized and those derived from fetal cells appeared consistently larger than those derived from adult cells. This difference in overall size reached after 7 d was confirmed in new series of experiments in which only one test cell of a defined phenotype and origin was added to each of a series of fibroblast-containing Matrigel cultures (Figure 5A). Immunohistochemical staining of the structures derived from single cells from the adult MRU-enriched EpCAM^+^CD49f^+^ fraction (blue gate in Figure 1C) showed that these consisted primarily of K5^+^, K14^+^ cells, and some p63^+^ cells (basal markers), with very few K8^+^ or K18^+^ cells (luminal markers) (Figure 5B). In contrast, we found single cells from the adult luminal progenitor-enriched EpCAM^++^CD49f^low/−^CD61^+^ fraction (solid red gate in Figure 1C) [28] generated structures that contained a mixture of cells expressing luminal and basal markers. By comparison, single cells from the fetal MRU-enriched EpCAM^++^CD49f^+^ fraction (Figure 1A) produced structures that most closely resembled those derived from the adult MRU-enriched EpCAM^+^CD49f^+^ cells in their predominant K5^+^, K14^+^, and p63^+^ cell content with a few progeny expressing the luminal markers, K8 and K18. Flow cytometric analysis of cell suspensions prepared individually from several of these structures showed that they were all similarly composed of homogeneous populations of EpCAM^+^CD49f^+^ cells with slightly higher levels of EpCAM expression in the structures derived from the originally EpCAM^++^CD49f^low/−^CD61^+^ adult (luminal progenitor-enriched) cells (Figure 5C).

In summary, all clonally generated structures examined after 7 d of growth consisted of cells with a “primitive” (CD49f^+^) phenotype and those derived from fetal MRU-enriched cells most closely resembled those derived from a functionally similar adult subset.

### Clonally Generated Structures Generated From Fetal Mammary Cells Contain More CFCs and MRUs Than Those Derived From Adult Cells

To determine the frequency of fetal and adult cells with structure-, CFC-, and MRU-generating activity and to compare their average outputs of CFCs and MRUs, we examined another series of 7-d Matrigel cultures initiated with single fetal and adult cells. In total, 163 cultures were initiated with single fetal (EpCAM^++^CD49f^+^) cells, 124 with single adult EpCAM^+^CD49f^+^ (basal) cells, and 100 with single adult EpCAM^++^CD49f^low/−^CD61^+^ (luminal) cells and visible structures were seen in 43%, 30%, and 18% of these, respectively (Table 1).

**Table 1:** Frequency of single cells that form structures and generate CFCs and MRUs from different subsets of fetal and adult mammary cells.

Endpoint Analyzed	Fetal	Basal	Luminal
Visible structures	43% (70/163)	30% (37/124)	18% (18/100)
Wells with CFCs	44% (72/163)	35% (44/124)	41% (41/100)
Structures with MRUs^a^	95% (20/21)	86% (12/14)	33% (3/9)
Wells with MRUs^b^	41% (95%×43%)	26% (86%×30%)	6% (33%×18%)

Values shown in brackets are the number of positive wells/number of wells assayed.

a Based on assessment of wells containing a visible structure.

b Calculated assuming MRUs would be found exclusively in wells containing visible structures.

The proportion of cultures initiated with a single fetal or adult basal cell that contained CFCs was very similar to the proportion that contained a visible structure (Table 1), consistent with the likelihood that all of the CFCs detected were present in these clonally derived structures. In contrast, we found CFCs in approximately twice as many cultures initiated with single adult luminal cells as contained a visible structure (41% versus 18%), consistent with the generally smaller size of these latter structures (Figure 5A). Notably, for all three input cell types, the frequency of cells that generated progeny with CFC activity in secondary assays was always higher (1.5- to 3-fold) than the frequency of input cells that displayed CFC activity directly (Figure 1B and 1D, Table 1).

**Figure 6:**
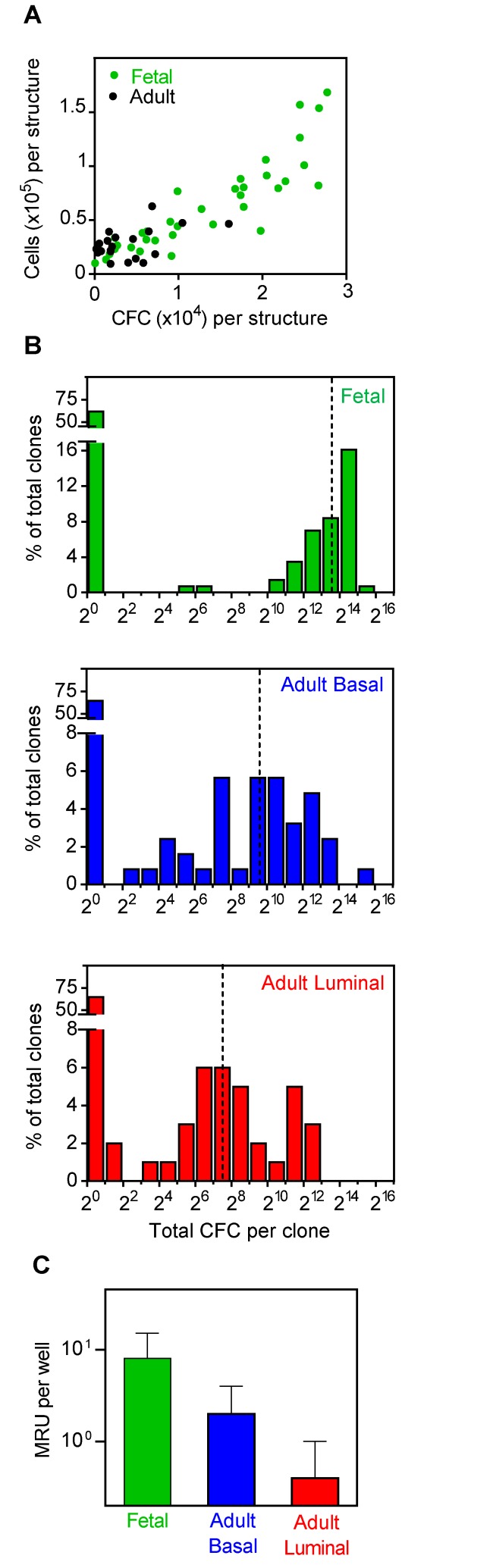
Comparison of CFC and MRU outputs in 7-d Matrigel cultures initiated with single fetal or adult mammary cells. (A) Correlation between the total number of cells retrieved from individually assessed 7-d cultures with the corresponding number of CFCs detected. (B) Distribution of clonal CFC outputs in 7-d cultures initiated with different types of input cells. The dotted line indicates the median values of positive clones: 1,000 for clones derived from basal adult cells (*n* = 44), 140 for clones from CD61^+^ adult luminal cells (*n* = 35), and 13,000 for clones from fetal cells (*n* = 55). (C) Comparison of MRU outputs determined by LDA in 7-d Matrigel cultures initiated with single adult and fetal mammary cells. The value for fetal cells is significantly higher than either of the adult values (*p*<0.01, Chi-square *p* value, pair-wise comparison of stem cell frequencies, ELDA, Table S7).

Measurement of the total number of cells as well as the total number of CFCs that had been produced in each of these clonal cultures showed that these output values were highly variable and correlated (Figure 6A and B) for all three sources of input cells. The highest CFC outputs were present in clones derived from fetal cells (up to >10^4^ CFCs per culture, median value ∼10-fold higher than for the basal cell clones, and almost 100-fold higher than for the luminal cell clones, Figure 6B).

**Figure 7:**
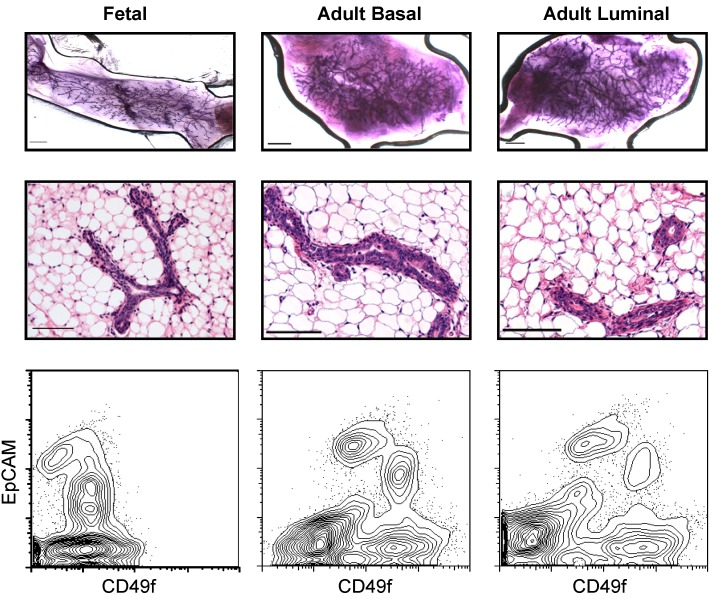
Regenerated glands produced *in vivo* from MRU generated *in vitro* from structures generated from single fetal, basal, or luminal cells. Whole mounts (upper panels) and sections (middle panels) of mammary glands produced in fat pads injected with cells from cultures initiated with single fetal cells (left panel), or single adult basal cells (middle panel) or single adult luminal cells (right panels). Scale bars, 100 µm (whole mounts) and 50 µm (sections). Lower panels show flow cytometry profiles of regenerated mammary glands from cultures initiated with single fetal cells (left panel), or single adult basal (middle panel) or six CD61^+^ luminal cells (right panels).

We also identified MRUs in the cultures initiated with single cells. The glands produced by these culture-generated MRUs were indistinguishable in morphology to those generated from freshly isolated cells (Figure 7). Most of the visible structures derived from the adult basal cells and some of those derived from the luminal progenitors also contained MRUs (86% and 33%, respectively, Table 1). If it is assumed that all MRUs produced in these cultures are associated with the formation of a visible structure, the frequency of adult mammary cells that can produce MRUs is at least 50-fold higher for the basal population (26% versus 0.3%) and >500-fold higher for the luminal cells (6% versus 0.01%) than the frequency of MRUs in the respective input populations, (Figure 1B and 1D, Tables 1 and S2).

The proportion of clonal structures produced from fetal cells that contained MRUs was even higher (95%, Table 1). This corresponds to a minimal frequency of fetal EpCAM^++^CD49f^+^ cells that can generate MRUs in our 7-d culture system that is 20-fold higher than the frequency of these initially isolated fetal cells that can be immediately detected as MRUs (41% versus 2%, Tables 1 and S4). Comparison of the clones generated from single fetal and adult cells showed that the average output of MRUs was significantly higher from the fetal cells (∼4-fold, *p*<0.01, Figure 6D and Table S7). Importantly, the MRUs generated from either single fetal or adult cells in this culture system not only regenerated normal-appearing glands in the cleared fat pad assay (Figure 7), the regenerated glands thus produced contained progeny MRUs that could be serially transplanted into secondary fat pads (6/7 positive fat pads from single fetal cells, and 9/11 positive fat pads from adult cells, Table S8).

### Comparison of the Transcriptomes of Fetal and Adult Mammary Cells with Different MRU-Generating Activity

**Figure 8:**
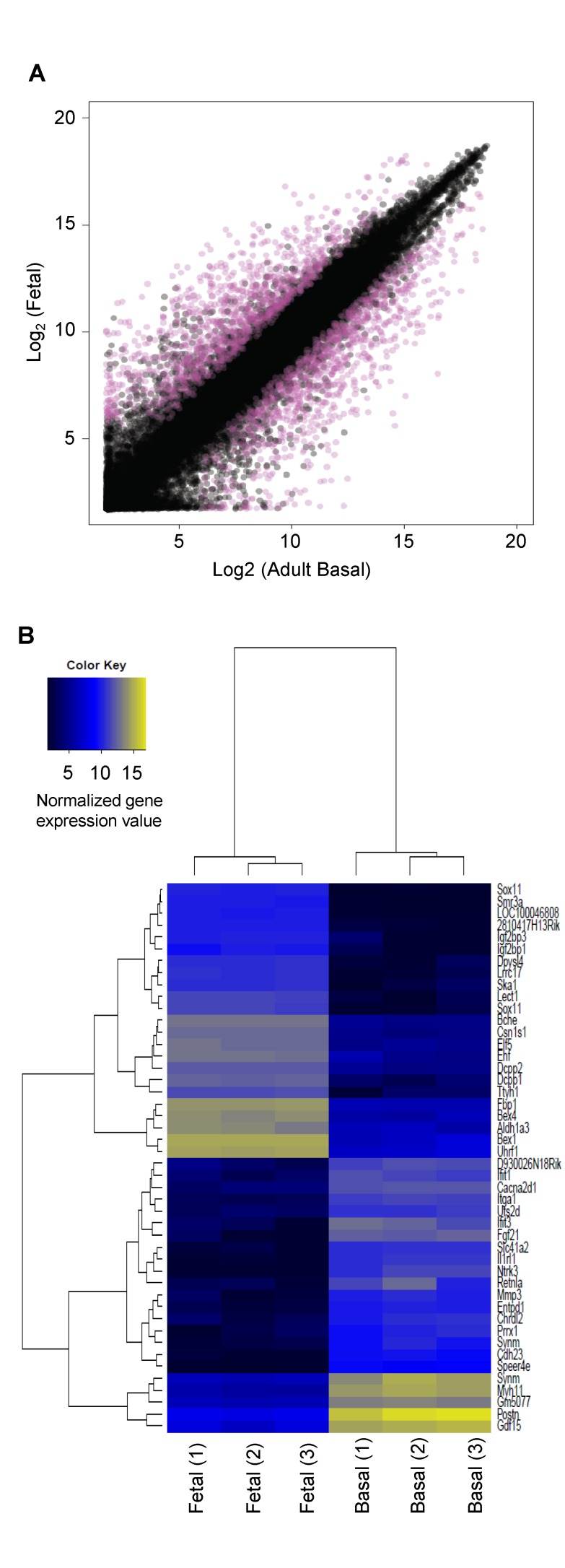
Microarray analysis of differentially expressed genes in MRU-enriched fetal and adult basal subsets of mammary cells. (A) Comparison of transcripts detected in Agilent arrays of extracts of E18.5 fetal (CD31^−^CD45^−^Ter119^−^EpCAM^++^CD49f^+^) and adult basal (CD31^−^CD45^−^Ter119^−^BP-1^−^EpCAM^+^CD49f^+^) cells. Expression values of both datasets are shown as log_2_-normalized values. Shown in pink are the genes (probes) identified as significantly differentially expressed (≥2-fold, *p*≤0.05). (B) Heat map of the top 1% of differentially expressed genes (probes) that were higher in the fetal dataset and the top 1% of differentially expressed genes (probes) that were higher in the adult basal dataset (shown as log_2_ normalized gene expression values from panel A).

As a first step toward elucidating the mechanism(s) contributing to the more potent MRU-generating activity of E18.5 fetal mammary cells as compared to their closest adult counterparts (the basal subset), we obtained global RNA profiles on both of these cell populations using Agilent arrays and compared them. A total of 2,262 genes (probes) showed significantly different levels of expression (≥2-fold, *p*≤0.05), with 1,206 genes up-regulated and 1,056 genes down-regulated in the fetal population (see Materials and Methods, Figure 8A and Table S9). Importantly, 937 (41%) of these differentially expressed transcripts overlapped with those identified in a previously published comparison of E18.5 fetal and adult MRU-enriched populations isolated using similar phenotypic markers [8], and with >90% concordance in the directionality of the differences seen.

The top 1% of differentially expressed genes (probes) that were higher in the fetal dataset and the top 1% of differentially expressed genes (probes) that were higher in the adult basal dataset are shown in Figure 8B. The most significant of these is *Sox11*, also noted by Spike et al. 2012 [8], and identified in a recent comparison of E12.5 mammary rudiments with adult mammary cells [29]. Additional genes of interest that we found are up-regulated in the E18.5 fetal cells include two that encode insulin growth factor 2 mRNA-binding proteins (*Igf2bp1* and *Igf2bp3*), which are let-7 miRNA targets broadly implicated in many tissue growth and metabolism networks operative during development [30]. Also in this group of highly up-regulated genes in fetal cells is *Elf5*, which encodes a member of the ETS family of transcription factors and has been reported to play a role in alveolar cell differentiation during pregnancy [31],[32]. Another is *Aldh1a3*, recently shown to be most highly expressed in the luminal progenitor compartment of normal adult human mammary cells [33]. Interestingly, we also confirmed *Ezh2* to be up-regulated in the fetal cells although to a slightly lower degree. Genes that were up-regulated in adult basal cells include *Mmp3*, previously shown to be involved in mammary gland morphogenesis [34], and *Myh11*, a myoepithelial cell-specific gene. Also in this latter group is *Itgα1*, an integrin α1 subunit, which forms a heterodimer with β1 and functions as a receptor for collagen [35].

## Discussion

There are four major findings emanating from these studies. First, we have identified profound differences in the differentiation stage-specific regulation of the proliferation and differentiation of isolated primitive mammary cells *in vitro*. Second, we show that the *in vitro* stimulation of latent regenerative activity is dependent on as yet undefined factors produced by fibroblasts that do not signal through the Wnt pathway, although this does not exclude the possibility of a co-operative role with Wnt signals [18]. Thirdly, we demonstrate a strong association of this cellular response with the formation of a visible structure in cultures initiated with single cells, although the magnitude of the response exhibited by individual cells varies over several orders of magnitude consistent with the operation of a self-renewal process that includes a stochastic component. Finally, we present the first and strong evidence of a marked superiority of the regenerative activity *in vitro* of primitive fetal as compared to adult mammary epithelial cells.

### Activation of CFC and MRU Potential in the Progeny of Cells That Lack This Activity

A key assumption in demonstrating an induction or activation of growth and differentiation activities not initially detectable is that the conditions used to elicit this activity are not simply suboptimal. Therefore, an important component of the present studies was an examination of the conditions used to detect CFC and MRU to ensure these were optimized. Current methods widely used to detect mammary progenitors that form colonies within 7 d in low-density 2D cultures at high efficiency include the addition of irradiated 3T3 cells [3]. We also adopted the use of a low O_2_ atmosphere [36],[37] and the addition of ROCKi [38], which we confirmed to be important modifications that selectively enhance the detection of adult basal CFCs and have a comparable effect on fetal CFCs. Similarly, several recent studies have indicated that the frequency of both adult and fetal mammary cells detectable as MRUs is enhanced if Matrigel is co-injected with the test cells [8],[19],[20],[39]. We confirmed this effect on both fetal and adult MRUs from C57Bl/6 mice and also implanted the recipients with E/P pellets to replicate the less consistently attainable enhancing effect of pregnancy on stimulating expansion of *in vivo* regenerated glands to enable their more facile visualization [21].

Importantly, even using these optimized assay conditions, we found that the frequency of fetal and adult mammary cells that could generate progeny with CFC and/or MRU activity in fibroblast-containing Matrigel cultures was always higher than the frequency of freshly isolated cells with directly demonstrable CFC and/or MRU activity. Moreover, the “induction/activation” process thus inferred could be elicited from all EpCAM^+^ subsets of cells examined and was true of fetal as well as adult mammary subsets. Particularly intriguing is the observation that MRUs could be derived from adult luminal progenitors, since these were previously thought to have irreversibly lost the bipotent, gland-generating activity of MRUs. However, it is interesting to note that evidence of persisting self-renewal of luminal cells has recently been reported based on the demonstrated presence in the adult of luminal cells derived from cells marked from postnatal day 1 by expression of the luminal marker, K8, although initiating the trace in cells expressing the basal marker, K14, in cells at E17 did result in the parallel detection of both basal and luminal phenotypes [40]. Similar studies using a different reporter, *Axin2*, have suggested that outcomes of such lineage-tracing experiments may vary depending on the type of gene used to “tag” different cell subsets [41], and have raised questions as to the unknown specificity in the embryo of lineage markers established in adult mammary epithelial tissue.

Adult ER^+^ or ER^−^ luminal cells were also recently shown to (re)activate bipotent features when “passaged” in collagen gels under the kidney capsule of transplanted mice [5]. The structures produced contained cells with basal features (p63 and smooth muscle actin) and MRU activity detected in subsequently injected cleared fat pads. Specific (HGF-mediated) activation of Met [23] and expression of Slug and Sox9 [20] have also been found to confer MRU activity on luminal cells. Slug alone was active on CD61^+^ luminal progenitors, whereas Slug in combination with Sox9 converted both CD61^+^ and CD61^−^ (differentiated luminal cells) into MRUs. Thus it is tempting to speculate that the acquisition of a cell surface luminal phenotype may sometimes precede the irreversible molecular “shut-down” of bipotency and self-renewal mechanisms that operate in MRUs that have a basal phenotype, and which can then be reactivated by factors produced by fibroblasts. Indeed evidence for such a model of alternative/latent stem cell populations in the skin [42] and crypt of the small intestine [43],[44] that can be reactivated under defined conditions has recently been reported.

### Fibroblasts Secrete Novel Factors Important for Stimulating the Acquisition of MRU and CFC Activity

Our finding that the addition of fibroblasts to Matrigel cultures strongly promotes the regenerative activity of cells in all subsets of adult and fetal mammary cells with MRU and CFC activity raises the important question as to the molecular mediators involved and the downstream mechanism by which the biological response is elicited. Recent reports have shown that induced Wnt signalling stimulates or is involved in qualitatively similar responses by developing [41] or adult [18] mammary or adult intestinal epithelial stem cells [26]. Our CM experiments showed that some of the effects of the added 3T3 fibroblasts in our cultures could be elicited by soluble factors that they release, but this could not be mimicked by the addition of Wnt3a, bFGF, HGF, or CSF-1. On the other hand, a contribution of Wnt signaling cannot be entirely ruled out. A minor effect was in fact obtained using one of two Wnt inhibitors, although this latter experiment could also reflect nonspecific effects of the inhibitor or a role of Wnt signaling in the production or release of different factors that may mediate the effects obtained or cooperate with other factors. Additional experiments to elucidate all of these possibilities and define the numbers and type of factors involved as well as their mode of action will clearly be of great interest and of potential relevance to understanding mammary cell oncogenesis. The availability of a rapid and robust clonal assay to discriminate active agents as now described should greatly accelerate such future investigations.

### Fetal Cells Have a Higher Induced Regenerative Activity Compared to Adult Cells

Our findings also provide the first evidence, to our knowledge, of a greater induction of MRU activity in fetal as compared to adult mammary epithelial cells demonstrable at the single-cell level. A higher self-renewal activity of fetal stem cells has been well-documented in the hematopoietic system [45],[46] and in the neural system [11],[15]. Several transcription factors and chromatin regulators that have been implicated in maintaining the unique properties of these fetal stem cells include *Sox17*, *Ezh2*, *Lin28*, and *Hmga2* [11]–[13],[15],[47]. We predict that similar intrinsic programs may be operative in fetal mammary cells, given the increased potency in the regenerative behaviour of fetal cells compared with adult mammary cells as assessed under identical conditions. Reported evidence of differences in gene expression of fetal and adult mouse mammary cells that are enriched in MRUs [8],[48] are corroborated by our own analysis of the gene expression profiles of fetal (EpCAM^++^CD49f^+^) and adult basal (EpCAM^+^CD49f^+^) B6 populations that are likely largely overlapping and shown here to be enriched in inducible as well as directly detectable MRUs. Indeed, our analyses show that ∼40% of the differentially expressed genes were also identified in the datasets analyzed by Spike et al. 2012 [8]. These include molecular regulators such as *Sox11*, *Igf2bp1-3*, *Elf5*, and *Ezh2*.

Taken together, our observations also appear relevant to growing evidence that some breast cancers may originate in luminal cells [23],[49], or expand from cells that have or may acquire luminal features [39]. The ease and rapidity with which this enormous proliferative potential of many normal mammary epithelial cells can be activated *in vitro* suggests that the mechanisms involved may also be targets of transforming events and act as covert contributors to the process of oncogenesis and clonal evolution in nascent breast cancers.

## Materials and Methods

### Mice

C57Bl6/J mice were used for all experiments and procedures approved by the Animal Care Committee of the University of British Columbia. Mice were considered E0.5 on the day of observed plug. All procedures involving mice were approved by the Animal Care Committee of the University of British Columbia.

### Cell Isolation

Mammary glands from 8–12-wk adult female and E18.5 fetal C57Bl6/J mice were digested overnight (adult) or for 1.5 h (fetal) at 37°C in DMEM/F12 medium containing 1 mg/ml collagenase A (Roche Diagnostics) and 100 U/ml hyaluronidase (Sigma) and single-cell suspensions obtained as described [3].

### 2D CFC Assays

Mammary cells and irradiated 3T3 fibroblasts were cultured for 6–7 d in media consisting of DMEM/F12 (3∶1, STEMCELL Technologies), 10% fetal bovine serum, 10 ng/ml EGF (Sigma), 1.8×10^−4^ M adenine (Sigma), 5 µg/ml insulin, 0.5 µg/ml hydrocortisone, 10^−10^ M cholera toxin (Sigma), and 10 µM Y-27632 (Reagents Direct). Cultures were incubated at 5% O_2_ unless indicated otherwise.

### *In vivo* Transplantations (MRU Assays)

These were performed as described [4] with the following modifications. We added 25% Matrigel (BD Biosciences) to the cell innoculum, and unless indicated otherwise, a silicone elastomer pellet containing 2 mg 17β-estradiol and 4 mg progesterone (E/P, Sigma) was implanted subcutaneously 3–4 wk posttransplant. Another 3–4 wk later, glands were fixed and stained. Outgrowths that contained a multiply branched structure were scored as positive. All MRU frequencies were calculated using ELDA software (http://bioinf.wehi.edu.au/software/elda/) [50].

### Matrigel Cultures

We combined 2.5×10^4^ irradiated 3T3 fibroblasts with the mammary cells in 200 µl of the same medium used for CFC assays, and the suspension was then placed on top of 50 µl of solidified (100%) Matrigel (Cat. No. 354234, BD Biosciences) previously added to each well of a 96-well plate. These cultures were then incubated at 37°C for 7 d in a humidified atmosphere containing 20% O_2_ without further medium addition or exchange. When other constituents were included, these were incorporated into the medium in which the test cells were initially suspended and, in all cases, the medium was not changed throughout the 7-d culture period. Additives tested were Wnt3a (R&D Systems), R-Spondin 1 (R&D Systems), XAV939 (Cellagen), mDKK1 (R&D Systems), bFGF (STEMCELL Technologies), mouse CSF-1 (STEMCELL Technologies), HGF (PeproTech), and 3T3 cell CM obtained by incubating the 3T3 cells in the 2D CFC assay medium for 48 h. To obtain a single-cell suspension from the 7-d cultures, 5 mg/ml dispase was first added for 1–1.5 h at 37°C and subsequently 0.25% trypsin/EDTA (both from STEMCELL Technologies) for 3–4 min at the end of which the cells were readily dissociated by pipetting. Transwell experiments were performed with scaled cultures in 24-well tissue culture plates with 1.0 µm pore size inserts.

### Flow Cytometry

Blocking of nonspecific antibody binding was performed by incubating cells for 10 min on ice in rat serum (Sigma) and anti-mouse CD16/32 Fc-gamma III/II Receptor antibody (Clone 2.4G2, STEMCELL Technologies). Mammary cells were depleted of hematopoietic, endothelial, and stromal cells using biotinylated antibodies to CD45 (clone 30-F11, Biolegend), erythroid cells (clone TER-119, Biolegend), CD31 (clone MEC 13.3, BD Pharmingen), and for adult cells only, also to BP-1 (clone 6C3, eBioscience), followed by streptavidin-eFluor780 (eBioscience) or streptavidin-phycoerythrin (PE, BD Pharmingen). Anti-CD49f-fluorescein isothiocyanate (FITC, clone GoH3, BD Pharmingen) and anti-CD326 (EpCAM)-AlexaFluor 647 (clone G8.8, Biolegend), and anti-CD61-PE (integrin β_3_) (clone 2C9.G2, BD Pharmingen) were used to isolate the fractions described. Cells were then exposed to 4′, 6-diamidino-2-phenylindole (DAPI) or propidium iodide (PI) to eliminate dead (DAPI^+^ or PI^+^) cells. The CD61^+^ fraction was isolated using fluorescence-minus-one controls, sorted from the adult luminal (CD45^−^CD31^−^Ter119^−^BP1^−^EpCAM^++^CD49f^+^) fraction. Cell sorting was performed using a FACSAria II or Influx II cell sorter (BD Biosciences).

### Immunohistochemistry

Single-cell derived structures in 96-well plates were fixed in 10% buffered formalin (Fisher) and subsequently washed in 70% ethanol. Structures were then individually removed and pooled for embedding in paraffin. We prepared 4 µm sections using Target Retrieval solution (DAKO), blocked using Cleanvision solution (Immunologic), and stained with an anti-cytokeratin 5 antibody (Clone AF138, Covance), anti-cytokeratin 8 antibody (ab 59400, Abcam), anti-cytokeratin 18 antibody (Clone E431-1, Millipore), anti-cytokeratin 14 antibody (LL002, Novocastra), and an anti-p63 antibody (Clone 4A4, BioCare Medical), and developed using the UltraVision ONE detection system (Fisher Scientific).

### Microarray Analysis

RNA was extracted using the Absolutely RNA Nanoprep kit (Agilent) from three biological replicates of purified CD31^−^CD45^−^Ter119^−^EpCAM^++^CD49f^+^ E18.5 fetal mammary cells and CD31^−^CD45^−^Ter119^−^BP-1^−^EpCAM^+^CD49f^+^ adult basal mammary cells. Total RNA quality was assessed with the Agilent 2100 bioanalyzer prior to microarray analysis. Samples with a RIN value of greater than or equal to 8.0 were deemed to be acceptable for microarray analysis. Samples were prepared following the Agilent One-Color Microarray-Based Exon Analysis Low Input Quick Amp WT Labeling v1.0. cRNA products were generated and hybridized to the Agilent SurePrint G3 Mouse GE 8x60K. Arrays were scanned with the Agilent DNA Microarray Scanner at a 3 µm scan resolution, and data were processed with Agilent Feature Extraction 11.0.1.1. Green processed signal was then quantile normalized with Agilent GeneSpring 12.0 and deposited at the gene expression omnibus (GSE46357).

To minimize multiple testing in comparing the data for the two sources of cells, we first eliminated probes that showed no/low activity in both adult basal and fetal cell datasets using a threshold for their elimination that was established by running a one-dimensional k-means algorithm (k = 3) on mean expression values for each probe in each dataset (three replicates each). The probes that fell in the lowest mean expression cluster in both datasets were thus identified and removed. This filtering left a total of 24,066 probes. Differential expression between the two datasets was then determined using the “lmFit” function in the R package “limma” and the Holm method to correct for the multiple testing methodology (R scripts available as Text S1). We thus identified 2,236 probes as showing a ≥2-fold difference between the two datasets using an adjusted *p* value of ≤0.05.

## Supporting Information

Figure S1 **Enhancing effects of low O_2_ and ROCKi on colony formation by fetal and adult cells.** CFC assays of unseparated E18.5 fetal cells (A, data pooled from 2–11 experiments) and unseparated adult cells (B, data pooled from three experiments). (C) CFC assays of purified basal and CD61^+^ luminal cells (data pooled from 3–18 experiments). All values shown are the mean ± SEM. Differences between results for 5% O_2_+ROCKi in all assays are significantly higher than corresponding results using 20% O_2_—ROCKi (*p*<0.05, one-way ANOVA with Bonferroni's multiple comparison test). (D) MRU assays of fetal and adult mammary epithelial cells in the absence and presence of an E/P pellet [results expressed per 100 EpCAM^++^ (fetal) or EpCAM^+^ (adult) cells]. Unseparated cells were used to estimate MRU numbers for adult (± E/P pellet, see Table S1, and purified EpCAM^++^ cells used to estimate MRU numbers for fetal (± E/P pellet), Table S3].
(TIF)

Figure S2 **Lack of effect of low O_2_ and minimal effect of ROCKi on CFC production in Matrigel cultures.** (A) Values shown are the fold-changes in CFCs detected in 7-d cultures initiated with fetal, adult basal, or adult luminal cells incubated at 5% or 20% O_2_ and assayed for CFC activity under optimal 5% O_2_ conditions as compared to input CFC numbers (data pooled from 1–4 experiments). (B) Similarly calculated changes in CFC numbers in cultures initiated with fetal, adult basal, or adult luminal cells maintained at 20% O_2_ in the presence or absence of ROCKi (data pooled from three experiments).
(TIF)

Table S1 **LDA of the enhanced MRU frequency in unseparated adult mammary cells tested in recipients given an E/P pellet.** Data pooled from five experiments.
(PDF)

Table S2 **LDA of the MRU frequency in different adult mammary basal and CD61^+^ luminal subsets.** Data pooled from two experiments.
(PDF)

Table S3 **LDA of the MRU frequency in unseparated E18.5 fetal mammary cells.** Data pooled from two experiments.
(PDF)

Table S4 **LDA of the MRU frequency in purified fetal mammary subsets.** Data pooled from four experiments.
(PDF)

Table S5 **LDA of the MRU frequency in 7-d Matrigel cultures initiated with fetal mammary cells.** Cultures were initiated with 325 (Exp 1) or 300 (Exp 2) unseparated fetal mammary cells (containing a calculated number of EpCAM^+^ cells) and co-cultured with irradiated 3T3 fibroblasts for 7 d. The contents of each well were then individually dissociated and assayed as described in Materials and Methods. The output MRU values are derived from the data pooled from both experiments.
(PDF)

Table S6 **LDA of the MRU frequency 7-d Matrigel cultures initiated with adult mammary cells.** Cultures were initiated with 300 unseparated adult mammary cells (containing a calculated number of EpCAM^+^ cells) and co-cultured with irradiated 3T3 fibroblasts for 7 d. The contents of each well were then individually dissociated and assayed as described in Materials and Methods. The output MRU values are derived from the data pooled from all three experiments.
(PDF)

Table S7 **LDA of the MRU frequency in 7-d Matrigel cultures initiated with single mammary epithelial cells from different sources.** Cells in cultures containing a visible structure derived from single EpCAM^++^ fetal, adult basal, or luminal cells were dissociated and assayed for MRUs as described in Materials and Methods.
(PDF)

Table S8 **Evidence that MRUs generated in 7-d Matrigel cultures produce daughter MRUs when they regenerate glands** ***in vivo*** **.** Matrigel cultures were initiated with fetal (seven single-cell cultures) or adult basal (four single-cell cultures, and one initiated with five cells) or adult luminal cells (five cultures each with six cells). Seven days later, a single cell suspension was prepared from each culture and the entire suspension injected into a cleared fat pad. Another 6–8 wk later, the 18 fat pads were individually dissociated into a single cell suspension and varying proportions of the harvested cells then transplanted into cleared fat pads of secondary mice. Another 6–8 wk later, these were scored for the presence or absence of a regenerated gland.
(PDF)

Table S9 **List of 2,262 transcripts detected in Agilent arrays of extracts of E18.5 fetal and adult basal cells in comparison with their regulation in Spike et al. 2012** [**8**] **.**
(XLS)

Text S1 **R scripts used to select significantly differentially expressed genes in the comparison of fetal and adult basal cell subsets analyzed.**
(TXT)

## Abbreviations

bFGF: basic fibroblast growth factor
CFC: colony-forming cell
CM: conditioned media
CSF-1: colony stimulating factor 1
DAPI: 4′, 6-diamidino-2-phenylindole
E: embryonic day
E/P: estrogen/progesterone
ER: estrogen receptor
FITC: fluorescein isothiocyanate
HGF: hepatocyte growth factor
K: keratin
LDA: limiting dilution analysis
MRU: mammary repopulating unit
PE: phycoerythrin
PI: propidium iodide
ROCKi: Rho-associated kinase inhibitor
