# Supplementary figures and images for: Developmental Changes in the in Vitro Activated Regenerative Activity of Primitive Mammary Epithelial Cells
Source: PLoS Biol. 2013 Aug 13;11(8):e1001630. doi: 10.1371/journal.pbio.1001630 (PMC3742452; doi:10.1371/journal.pbio.1001630)

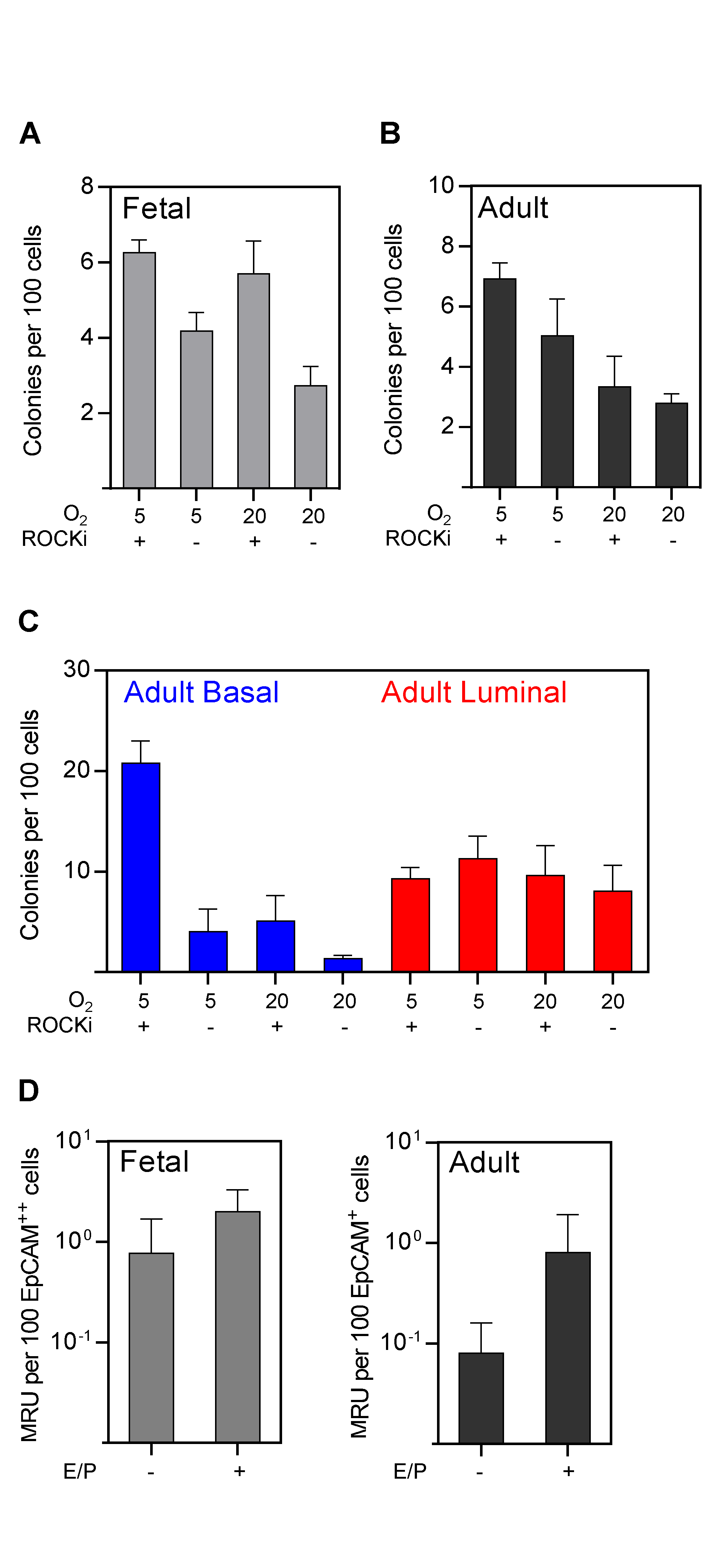

Supplement: Figure S1 — Enhancing effects of low O2 and ROCKi on colony formation by fetal and adult cells. CFC assays of unseparated E18.5 fetal cells (A, data pooled from 2–11 experiments) and unseparated adult cells (B, data pooled from three experiments). (C) CFC assays of purified basal and CD61+ luminal cells (data pooled from 3–18 experiments). All values shown are the mean ± SEM. Differences between results for 5% O2+ROCKi in all assays are significantly higher than corresponding results using 20% O2—ROCKi (p<0.05, one-way ANOVA with Bonferroni's multiple comparison test). (D) MRU assays of fetal and adult mammary epithelial cells in the absence and presence of an E/P pellet [results expressed per 100 EpCAM++ (fetal) or EpCAM+ (adult) cells]. Unseparated cells were used to estimate MRU numbers for adult (± E/P pellet, see Table S1, and purified EpCAM++ cells used to estimate MRU numbers for fetal (± E/P pellet), Table S3]. (TIF) [file pbio.1001630.s001.tif]

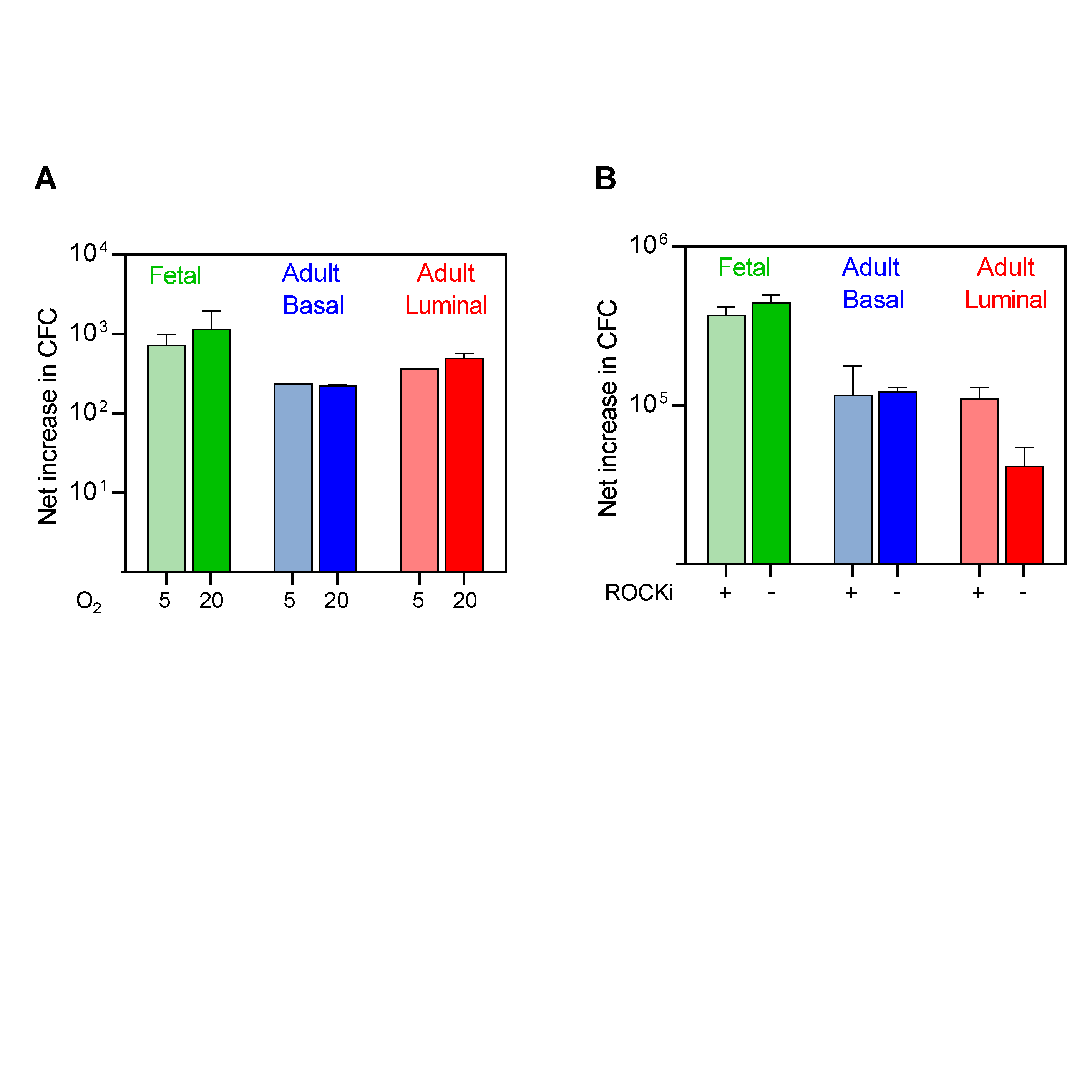

Supplement: Figure S2 — Lack of effect of low O2 and minimal effect of ROCKi on CFC production in Matrigel cultures. (A) Values shown are the fold-changes in CFCs detected in 7-d cultures initiated with fetal, adult basal, or adult luminal cells incubated at 5% or 20% O2 and assayed for CFC activity under optimal 5% O2 conditions as compared to input CFC numbers (data pooled from 1–4 experiments). (B) Similarly calculated changes in CFC numbers in cultures initiated with fetal, adult basal, or adult luminal cells maintained at 20% O2 in the presence or absence of ROCKi (data pooled from three experiments). (TIF) [file pbio.1001630.s002.tif]
