# Supplementary material for: Developmental Changes in the in Vitro Activated Regenerative Activity of Primitive Mammary Epithelial Cells
Source: PLoS Biol. 2013 Aug 13;11(8):e1001630. doi: 10.1371/journal.pbio.1001630 (PMC3742452; doi:10.1371/journal.pbio.1001630)
Supplement: Table S1 — LDA of the enhanced MRU frequency in unseparated adult mammary cells tested in recipients given an E/P pellet. Data pooled from five experiments. (PDF) [file pbio.1001630.s003.pdf]

Table S1.

Use of E/P pellet	Cell dose	Positive fat pads/ total	MRU frequency (95% CI)
+	1,000	7/8	1/480 (1/200 – 1/1,200)
-	2,500	1/4	1/5,000 (1/2,400 – 1/10,600)
	2,000	0/3	
	1,000	6/20	
	500	0/7	
