# Supplementary material for: Developmental Changes in the in Vitro Activated Regenerative Activity of Primitive Mammary Epithelial Cells
Source: PLoS Biol. 2013 Aug 13;11(8):e1001630. doi: 10.1371/journal.pbio.1001630 (PMC3742452; doi:10.1371/journal.pbio.1001630)
Supplement: Table S2 — LDA of the MRU frequency in different adult mammary basal and CD61+ luminal subsets. Data pooled from two experiments. (PDF) [file pbio.1001630.s004.pdf]

Table S2.

Use of E/P pellet	Fraction	Cell dose	Positive fat pads/total	MRU frequency (95% CI)
+	EpCAM^{+}CD49f^{+}	250	3/5	1/290
	(Basal)	50	1/6	(1/100 – 1/780)
		10	0/5	
+	EpCAM^{++}CD49f$^{low/-}$ CD61^{+}	5,000	3/7	1/8,900
	(Luminal)			(1/2,800 - 1/28,000)
