# Supplementary material for: Developmental Changes in the in Vitro Activated Regenerative Activity of Primitive Mammary Epithelial Cells
Source: PLoS Biol. 2013 Aug 13;11(8):e1001630. doi: 10.1371/journal.pbio.1001630 (PMC3742452; doi:10.1371/journal.pbio.1001630)
Supplement: Table S3 — LDA of the MRU frequency in unseparated E18.5 fetal mammary cells. Data pooled from two experiments. (PDF) [file pbio.1001630.s005.pdf]

Table S3.

Use of E/P pellet	Cell dose	Positive fat pads/ total	MRU frequency (95% CI)
-	25,000	2/2	1 /1,000
	5,000	2/3	(1/500 - 1/2,500)
	1,000	5/7	
	500	3/4	
	100	1/2	
