# Supplementary material for: Developmental Changes in the in Vitro Activated Regenerative Activity of Primitive Mammary Epithelial Cells
Source: PLoS Biol. 2013 Aug 13;11(8):e1001630. doi: 10.1371/journal.pbio.1001630 (PMC3742452; doi:10.1371/journal.pbio.1001630)
Supplement: Table S4 — LDA of the MRU frequency in purified fetal mammary subsets. Data pooled from four experiments. (PDF) [file pbio.1001630.s006.pdf]

Table S4.

Use of E/P pellet	Fraction	Cell dose	Positive fat pads/total	MRU frequency (95% CI)
+	EpCAM^{-}	10,000	0/8	<1/75,000
+	EpCAM^{+}	500	6/8	1/360
				(1/150 - 1/860)
+	EpCAM^{++}	250	4/4	1/50
		50	8/15	(1/30 - 1/100)
		10	3/11	
-	EpCAM^{++}	2,000	4/4	1/130
		500	4/4	(1/60 - 1/280)
		200	0/2	
		100	4/4	
		50	1/4	
		10	1/6	
