# Supplementary material for: Developmental Changes in the in Vitro Activated Regenerative Activity of Primitive Mammary Epithelial Cells
Source: PLoS Biol. 2013 Aug 13;11(8):e1001630. doi: 10.1371/journal.pbio.1001630 (PMC3742452; doi:10.1371/journal.pbio.1001630)
Supplement: Table S6 — LDA of the MRU frequency 7-d Matrigel cultures initiated with adult mammary cells. Cultures were initiated with 300 unseparated adult mammary cells (containing a calculated number of EpCAM+ cells) and co-cultured with irradiated 3T3 fibroblasts for 7 d. The contents of each well were then individually dissociated and assayed as described in Materials and Methods. The output MRU values are derived from the data pooled from all three experiments. (PDF) [file pbio.1001630.s008.pdf]

Table S6.

Exp No.	EpCAM$^+$ cells/well	Input MRU (95% CI)	Fraction of well/fat pad	Positive fat pads/total	Output MRU/well (95% CI)	MRU/ 100 input EpCAM$^+$ cells
1	80	0.6<br>(0.2 - 1.6)	1/6th<br>1/20th	4/4<br>1/4	11<br>(5 - 23)	14<br>(6 - 29)
2	80	0.6<br>(0.2 - 1.6)	1/20th	1/3		
3	80	0.6<br>(0.2 -1.6)	1/20th	1/3		
