# Supplementary material for: Developmental Changes in the in Vitro Activated Regenerative Activity of Primitive Mammary Epithelial Cells
Source: PLoS Biol. 2013 Aug 13;11(8):e1001630. doi: 10.1371/journal.pbio.1001630 (PMC3742452; doi:10.1371/journal.pbio.1001630)
Supplement: Table S7 — LDA of the MRU frequency in 7-d Matrigel cultures initiated with single mammary epithelial cells from different sources. Cells in cultures containing a visible structure derived from single EpCAM++ fetal, adult basal, or luminal cells were dissociated and assayed for MRUs as described in Materials and Methods. (PDF) [file pbio.1001630.s009.pdf]

Table S7.

Cell source	Fraction of well/fat pad	Positive fat pads/total	Output MRU/well (95% CI)
Fetal	100%	6/7	8
	60%	7/7	(5 - 15)
	10%	15/18	
	1%	1/3	
Adult Basal	100%	12/14	2
	10%	2/4	(1 - 4)
Adult Luminal	100%	3/9	0.4
	10%	0/1	(0.1 - 1)
