# Supplementary material for: Developmental Changes in the in Vitro Activated Regenerative Activity of Primitive Mammary Epithelial Cells
Source: PLoS Biol. 2013 Aug 13;11(8):e1001630. doi: 10.1371/journal.pbio.1001630 (PMC3742452; doi:10.1371/journal.pbio.1001630)
Supplement: Table S8 — Evidence that MRUs generated in 7-d Matrigel cultures produce daughter MRUs when they regenerate glands in vivo . Matrigel cultures were initiated with fetal (seven single-cell cultures) or adult basal (four single-cell cultures, and one initiated with five cells) or adult luminal cells (five cultures each with six cells). Seven days later, a single cell suspension was prepared from each culture and the entire suspension injected into a cleared fat pad. Another 6–8 wk later, the 18 fat pads were individually dissociated into a single cell suspension and varying proportions of the harvested cells then transplanted into cleared fat pads of secondary mice. Another 6–8 wk later, these were scored for the presence or absence of a regenerated gland. (PDF) [file pbio.1001630.s010.pdf]

Table S8.

Cell source	No. of starting cells / culture	No. of cultures tested	% of 1°regenerated gland / 2°fat pad	No. of positive 2°fat pads
Fetal	1	1	50%	1
	1	3	30%	3
	1	3	10%	2
**Total fetal cultures**		**7**		**6**
Adult basal	1	2	30%	1
	1	1	10%	1
	1	1	5%	0
	5	2	30%	2
Adult luminal	6	4	100%	4
		1	30%	1
**Total adult cultures**		**11**		**9**
